# A review and analysis of cryptosporidiosis outbreaks in New Zealand

**DOI:** 10.1017/S0031182023000288

**Published:** 2023-06

**Authors:** Juan C. Garcia-R, David T. S. Hayman

**Affiliations:** Molecular Epidemiology and Public Health Laboratory, Hopkirk Research Institute, Massey University, Private Bag 11–222, Palmerston North, New Zealand

**Keywords:** Cryptosporidiosis, molecular typing, New Zealand, outbreaks, surveillance, TIDE analysis

## Abstract

*Cryptosporidium* is a leading global cause of diarrhoea with many reported outbreaks related to water and zoonotic transmission. This study summarizes data from Public Health Surveillance reports since 2010 in New Zealand to describe exposures associated with human diarrhoea outbreaks caused by *Cryptosporidium*. We investigate the species and subtypes of cases involved in some of the outbreaks to elucidate transmission routes and the predominant aetiological agents of cryptosporidiosis. For the period 2010–2017, 318 cryptosporidiosis outbreaks were reported in New Zealand resulting in 1634 cases and 20 hospitalizations. The most important mode of transmission was person-to-person (primary infections and secondary or close contacts infections), relating to 260 outbreaks and 1320 cases, followed by 113 outbreaks associated with animals, resulting in 436 human cases. From 2018 to 2021, there were 37 cryptosporidiosis outbreaks associated with 324 cases. We identified the subtypes by using polymerase chain reaction targeting the gp60 gene and the likelihood of mixed subtype infections with the Tracking of Indels by DEcomposition (TIDE) algorithm. Subtype families Ib and Ig of *Cryptosporidium hominis* and IIa and IId of *Cryptosporidium parvum* were found among cases; however, *C. hominis* subtypes occurred in 8 of the 11 outbreaks reviewed where molecular data were available. Examination of the chromatograms showed no mixed subtype infections in the samples assessed. Subtyping data need to be routinely incorporated into national surveillance programmes to better understand the epidemiology, sources, transmission and extent of cryptosporidiosis outbreaks in New Zealand. Our study highlights the value of integrating epidemiological information and molecular typing to investigate and manage clusters of cryptosporidiosis cases.

## Graphical abstract

**Figure:**
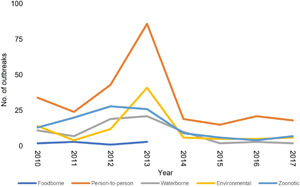

## Introduction

Cryptosporidiosis is a diarrhoeal disease caused by *Cryptosporidium* species, ubiquitous enteric parasites that can infect a variety of hosts, including humans (Chalmers and Giles, 2010; Xiao, 2010). The global pooled prevalence of *Cryptosporidium* infection in people is estimated at 7.6% (Dong *et al*., 2020). In high-income countries, the reported prevalence ranges from 0.1 to 14.1% with an average estimate of 4.3%, whilst in low-income countries the reported prevalence ranges from 1.3 to 31.5% and an average estimate of 10.4% (Fayer, 2004; Dong *et al*., 2020; Liu *et al*., 2020). Asymptomatic carriage is usually low, particularly in high-income countries, and has been reported to range from 0 to 4% (Enserink *et al*., 2015; Reh *et al*., 2019). Further, while it has been reported that *Cryptosporidium* is present in 1% of stools of immunocompetent individuals in high-income countries, the pathogen is detected in 5–10% of stools of people in low-income countries (Checkley *et al*., 2015). However, the diagnosis of pathogens causing gastroenteritis may be as low as 1% of true prevalence in high-income countries and even lower in low-income countries (Scallan *et al*., 2011).

*Cryptosporidium* is responsible for several well-documented food- and waterborne as well as occupational outbreaks (Karanis *et al*., 2006; Baldursson and Karanis, 2011; Widerström *et al*., 2014; Efstratiou *et al*., 2017; Hancock-Allen *et al*., 2017). A cryptosporidiosis outbreak can be defined as 2 or more cases epidemiologically linked to a common source by location and time of exposure (Gharpure *et al*., 2019). Transmission can occur by ingestion of contaminated water or food and through contact with the feces of infected people or animals (Fayer *et al*., 2000). There were 120 *Cryptosporidium* waterborne outbreaks reported worldwide from 2004 to 2010 (Baldursson and Karanis, 2011). Outbreaks are more commonly notified in high-income countries due to better notification and surveillance systems (Efstratiou *et al*., 2017). Waterborne outbreaks can be very large with some of the largest reported from Europe and the USA. For example, a waterborne cryptosporidiosis outbreak in Milwaukee, USA, affected more than 400 000 people during 2 months in 1993, with an associated cost estimated over US$96 million (Corso *et al*., 2003). In Sweden, 2 waterborne outbreaks were reported in 2010 and 2011 affecting about 47 000 people (Widerström *et al*., 2014). Between 2004 and 2014, 28.5% of the worldwide waterborne cryptosporidiosis outbreaks were reported in New Zealand (Efstratiou *et al*., 2017).

Cryptosporidiosis has been a notifiable disease in New Zealand since 1996 (Learmonth *et al*., 2004). The reported incidence of the disease in humans from New Zealand is very high, even though it is estimated that only 2% of acute gastrointestinal illnesses, including cryptosporidiosis, are notified (Snel *et al*., 2009*a*, 2009*b*). Compared to other high-income countries, New Zealand reports between 26.1 and 32.3 new cases per 100 000 population per year, whilst Australia reports 12.8 per 100 000 population, England and Wales 8.0 per 100 000, USA 2.9 per 100 000, and Canada 2.7 per 100 000 (Learmonth *et al*., 2004; Lal *et al*., 2015; Garcia-R *et al*., 2020). Cryptosporidiosis incidence in New Zealand is suggested to be increasing partly because of outbreaks from untreated water and farming. However, the number of cases involved in most outbreaks is small compared to outbreaks in other high-income countries (Stefanogiannis *et al*., 2001; Snel *et al*., 2009*b*; Grinberg *et al*., 2011). This might not only reflect high disease burden, but also effective surveillance and primary health care reporting.

Here, we use reports from outbreaks occurring since 2010 submitted by the Institute of Environmental Science and Research Ltd (ESR) to the Public Health Surveillance website (https://surv.esr.cri.nz/index.php) to highlight emerging aspects of cryptosporidiosis in New Zealand. We also employed molecular data records collected by our laboratory to describe epidemiologic features and gain a better understanding of species and subtypes involved in cryptosporidiosis outbreaks in New Zealand.

## Methods

We used the cryptosporidiosis outbreak data provided by the Public Health Surveillance website (https://surv.esr.cri.nz/index.php) over the years 2010–2021. We also extracted data on the modes of transmission and hospitalized cases when available from the Public Health Surveillance website. Before 2017, the data about hospitalized cases and modes of transmissions were individually documented for each pathogen, including *Cryptosporidium*, but since 2018 this information was supplied for all notifiable diseases, not allowing us to collect the data for outbreaks caused by *Cryptosporidium* only. Sources and modes of transmission are reported following the ESR Guidelines for the Investigation and Control of Disease Outbreaks (Institute of Environmental Science & Research Limited, 2012).

Our laboratory, the Protozoa Research Unit at Hopkirk Research Institute (Massey University), has used molecular characterization of *Cryptosporidium*-positive stool samples from symptomatic humans visiting private and public general practitioners (GP) throughout New Zealand under a Ministry of Health contract for genetic surveillance. Some of the samples sent to our lab are from cases associated with outbreaks. We collected the genetic variant(s) involved in 87 human samples from historic cases of cryptosporidiosis outbreaks occurring between 2010 and 2021 (Table 1) previously analysed by extracting DNA and polymerase chain reaction (PCR) molecular typing of the gp60 gene. The sequences have been previously submitted to GenBank (Garcia-R *et al*., 2017, 2020). High quality and accessible archive of gp60 forward chromatograms generated in our laboratory using Sanger sequencing for 23 samples involved in the outbreaks were used in the Tracking of Indels by DEcomposition (TIDE) algorithm (Dettwiler *et al*., 2021) to identify mixed subtype infections and stutter artefacts. We re-investigate the chromatograms generated by Sanger sequencing in 6 outbreaks (Taranaki 2013 and 2021, Auckland 2015 and 2017, Wellington 2013 and Blenheim 2017) to identify shifts in the target sequences and the likelihood of multiplicity of infections using the TIDE algorithm. Forward strand chromatograms from other outbreaks were not located or have low quality, making them unsuitable for the TIDE analysis. We follow Dettwiler *et al*. (2021) in applying a conservative threshold for differentiating mixed infections from stutter (a reverse stutter filter of 15% of sequences for N–3 and 8% of sequences for N–6).

**Table 1.** Cryptosporidiosis outbreaks between 2010 and 2021 reported in this study

Year	Location	Number of cases	Species	Genotype	Source of outbreak
2010	Auckland	7	*C. hominis*	IbA10G2	Swimming pool
	Christchurch	17	*C. hominis*	IbA10G2	Unknown
2013	Hawke's Bay	22	*C. hominis*	IgA17	Swimming pool
	Waikato	5	*C. hominis*	IbA10G2 and IgA17	Unknown
	Wellington	5	*C. hominis*	IbA10G2 and IgA17	Unknown
	Taranaki	3	*C. hominis*	IbA10G2	Unknown
2015	Auckland	6	*C. parvum*	IIaA18G3R1	Raw milk
2017	Auckland	9	*C. hominis*	IbA10G2 and IgA20	Childcare centre
	Blenheim	3	*C. hominis* and *C. parvum*	IbA10G2 and IIdA24G1	Swimming pool
2021	Taranaki	6	*C. parvum*	IIaA18G3R1 and IIaA19G4R1	Raw milk
	Taranaki	4	*C. parvum*	IIaA18G3R1 and IIaA19G4R1	Raw milk

## Results

**Fig. 1.**
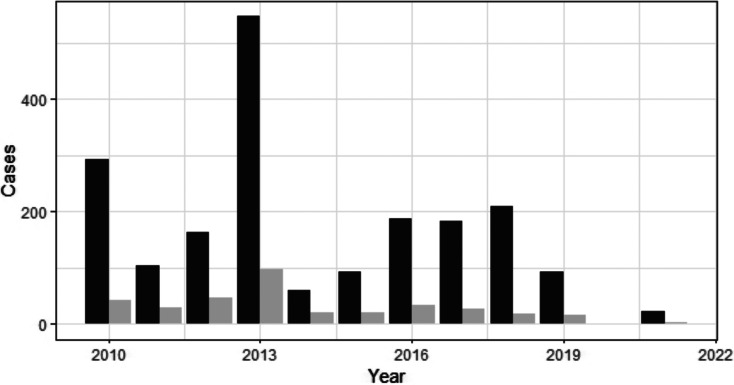
Reported cryptosporidiosis outbreaks (grey) and the number of associated cases with outbreaks (black) by year. The decrease in cryptosporidiosis cases since 2020 is associated with Covid-19 restrictions (Knox *et al*., 2021).

Cryptosporidiosis outbreak-associated cases from 2010 to 2021 show distinct peaks in various years with the highest number of notifications in 2013 (*n* = 547), followed by 2010 (*n* = 294) and 2018 (*n* = 209) (Fig. 1). For the period 2010–2017, 20 hospitalizations and multiple modes of transmission associated with different risk factors were reported. The most important mode of transmission was person-to-person (primary infections and secondary or close contacts infections), related to 260 outbreaks and 1320 cases, followed by 113 outbreaks associated with animals, resulting in 436 human cases (Table 2). From 2018 to 2021, there were 37 cryptosporidiosis outbreaks associated with 324 cases (Fig. 1).

**Table 2.** Cryptosporidiosis outbreaks by mode of transmission and exposure from 2010 to 2017

Transmission mode	Outbreaks	Cases
**All modes**	**318**	**1634**
Person-to-person	260	1320
Zoonotic	113	436
Environmental	93	746
Waterborne	75	224
Foodborne	10	29

Note that outbreaks can be associated with different modes of transmission when evidence to implicate 1 specific contributing factor is complicated or insufficient.

**Fig. 2.**
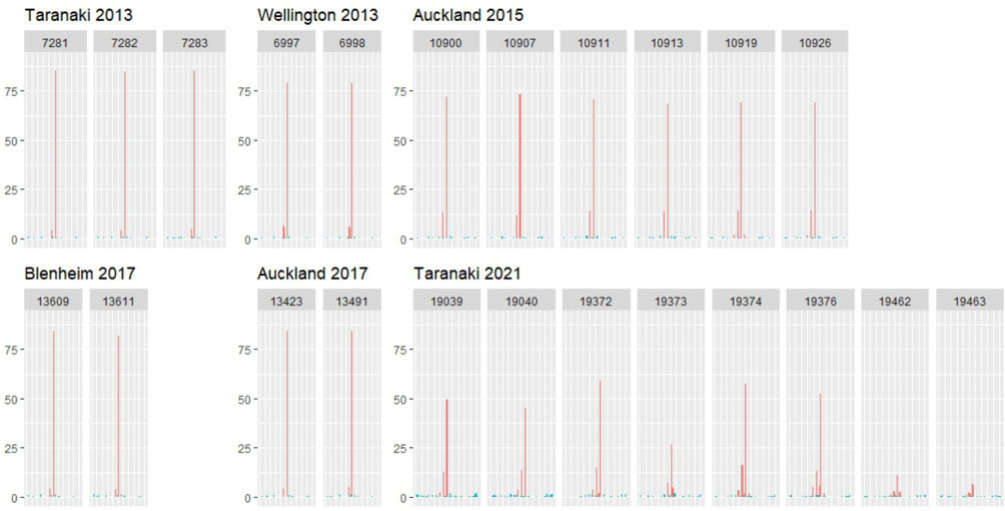
Indel spectrums from TIDE analyses of sequences from different outbreaks in New Zealand. Outbreak location, year of collection and sample numbers are shown. *Y*-axis is the percentage of sequences, *X*-axis the relative position and bar colours show *P* value <0.001 (red) or not (blue). Underlying codon-size variations (e.g. −6 nt and −3 nt) suggest stutter artefacts, not coinfection with multiple strains in the sample.

Our laboratory has been involved in the molecular characterization of samples associated with 11 cryptosporidiosis outbreaks. Two outbreaks during March 2010 that occurred in Auckland and Christchurch showed *C. hominis* 1bA10G2 as the most predominant variant. The outbreak in Auckland was associated with a contaminated swimming pool (Table 1). We also describe the molecular epidemiology of 4 outbreaks during the summer/autumn period of 2013 in Hawkes Bay, Waikato, Wellington and Taranaki regions. The outbreak in Hawkes Bay was associated with *C. hominis* IgA17, the outbreak in Taranaki with *C. hominis* 1bA10G2, whilst the outbreaks in Wellington and Waikato showed both subtypes. In 2015, we were involved in an outbreak in Auckland related to the consumption of raw milk. Samples were positive for *C. parvum* IIaA18G3R1. In May 2017, we identified *C. hominis* subtypes IgA20 and IbA10G2 in an outbreak in Auckland. In June 2017, a swimming pool complex in Blenheim was linked to a cryptosporidiosis outbreak with *C. hominis* subtype IbA10G2 and *C. parvum* subtype IIdA24G1. In May and September 2021, 2 outbreaks associated with the consumption of raw milk in Taranaki were found to be caused by both *C. parvum* IIaA18G3R1 and IIaA19G4R1 subtypes. All gp60 chromatograms analysed using the TIDE algorithm (Fig. 2) were found with stutter artefacts without potential coinfections with different subtypes within individual people. We mainly found reverse N–3 (main allele minus 3 nucleotides) and N–6 (allele minus 6 nucleotides) stutter artefact peaks in TIDE. Curiously, the outbreak in Wellington 2013 showed peaks at N–2 and N–3 (Supplementary Fig. S1), and multiple peaks at varying positions on samples in the outbreak at Taranaki 2021 (Table S1).

## Discussion

*Cryptosporidium* is one of the most common pathogens causing infectious disease outbreaks in New Zealand, along with *Giardia* and norovirus. From 2010 to 2017, *Cryptosporidium* was implicated in 318 (6%) of the 5230 infectious disease outbreaks reported in New Zealand. In 2013, *Cryptosporidium* was the second most reported agent causing gastrointestinal diseases, accounting for 15% of outbreaks (98/652) and 7.7% of associated cases (547/7137) (https://surv.esr.cri.nz/surveillance/annual_surveillance.php). During 2013 there were a high number of cryptosporidiosis cases (1348 cases in total), many (547 cases) associated with outbreaks (Garcia-R *et al*., 2020).

Cryptosporidiosis outbreaks in New Zealand have been reported from farm environments, food sources, contaminated water and following anthroponotic transmission. Our molecular work identified 8 of the 11 outbreaks to be caused by *C. hominis* subtypes. This and the high incidence of person-to-person transmission as a mode of transmission indicates that *C. hominis* is the most common species in outbreaks and dominated by anthropogenic transmission, which differs to surveillance cases where *C. parvum* is more dominant (Garcia-R and Hayman, 2017; Garcia-R *et al*., 2017, 2020). The importance of *C. hominis* in person-to-person transmission can also be inferred by the absence of *C. hominis* cases reported and the lack of *C. hominis* outbreaks in 2020 during the Covid-19 restrictions in New Zealand (Knox *et al*., 2021). Limitations on international travel and nationwide lockdown limited person-to-person transmission, but not human–animal contacts, evidenced by continued *C. parvum* cases. Transmission rates in the UK have also been found to be higher for *C. hominis* compared with *C. parvum* and other species, mainly in households, child-care facilities, nursing homes and schools (McKerr *et al*., 2022).

Waterborne and foodborne outbreaks of cryptosporidiosis have occurred less frequently than person-to-person transmission. However, this may be related to an absence of notification and a lack of investigation rather than to truly different epidemiological situations. Sporadic cases associated with water and food are likely unrecognized. *Cryptosporidium* has been responsible for numerous waterborne outbreaks worldwide (Baldursson and Karanis, 2011; Efstratiou *et al*., 2017). Outbreaks linked to recreational waters, especially swimming pools, have increased in the UK and the USA (Cacciò and Chalmers, 2016). In the USA, a review of 444 cryptosporidiosis outbreaks resulting in 7465 cases between 2009 and 2017 revealed that contaminated recreational water (treated and untreated) was responsible for 60% (4495/7465) of cases, 38% of outbreaks and 64% (*n* = 186) of hospitalizations (Gharpure *et al*., 2019). In addition, treated recreational water-associated outbreaks increased by ~14% per year between 2009 and 2016 (Gharpure *et al*., 2019). In Australia, a review between 2001 and 2007 of waterborne outbreaks identified 78% attributed to recreational water, 98% of them caused by *Cryptosporidium* (Dale *et al*., 2010). Recreational water-associated outbreaks worldwide have predominantly been caused by *C. hominis* (Causer *et al*., 2006; Cope *et al*., 2015; Chalmers *et al*., 2019; Zahedi and Ryan, 2020; Ryan *et al*., 2022). For instance, *C. hominis* is responsible for all recreational water-associated outbreaks in Australia and, similarly to the UK and NZ, it has been found to have a higher infectivity for humans than *C. parvum* (Ryan *et al*., 2022).

*Cryptosporidium hominis* subtype IbA10G2 was found in outbreaks in Auckland, Christchurch and Taranaki and it is highly prevalent in outbreak cases in Europe, Australia and the USA (Chalmers *et al*., 2008; Ng *et al*., 2010). Subtype IgA17 of *C. hominis* was involved in a large outbreak in Hawkes Bay (reported here) and other subsequent outbreaks in the North Island of New Zealand during the summer and autumn of 2013. Before 2013, the subtype Ig was not previously registered in New Zealand (Garcia-R *et al*., 2017, 2020). This subtype was relatively rare overseas, but it has been isolated from human cases in Australia (Ng *et al*., 2010). It might indicate that IgA17 was introduced in New Zealand from Australia with subsequent widespread disseminations from the Hawkes Bay to other regions, potentially following several swimming pool-related outbreaks. *Cryptosporidium parvum* was less commonly found in waterborne outbreaks and mainly associated with consumption of raw milk. Nonetheless, subtype IIaA18G3R1, identified in some outbreaks in this study, has been reported as a cause of waterborne outbreaks of human cryptosporidiosis in Europe and Australia (Glaberman *et al*., 2002; Chalmers *et al*., 2005). Zoonotic and environmental transmissions account for almost half of *Cryptosporidium* outbreaks, possibly linked to farming and contamination of drinking and recreational water (Phiri *et al*., 2020; Grout *et al*., 2022).

Variability in the sequence of the gp60 gene has been useful for inferring transmission routes. However, different gp60 subtypes can be linked to the same outbreak, which may indicate a coinfection of multiple subtypes, or an artefact caused by DNA polymerase slippage during the PCR elongation step. TIDE allowed us to differentiate between stutter effects and coinfection by multiple subtypes in the samples. The TIDE analyses in our study identified stutters within outbreak sample sequences but did not support evidence of mixed subtype infections. Most effects were N-3 or, less frequently, N−6. For example, the outbreak in Taranaki 2013 was caused by *C. hominis* subtype IbA10G2 and there was evidence of N−3 stutter. However, the outbreak in Wellington 2013 showed peaks at N−2 (unexpected result because they should be a multiple of 3) and N−3 and the outbreak in Taranaki 2021 presented multiple peaks. None reached the level of support suggested by Dettwiler *et al*. (2021); however, further analyses such as metabarcoding might identify mixed infections, including mixed family subtypes and species (Garcia-R *et al*., 2021; Ogbuigwe *et al*., 2022).

Although cryptosporidiosis in New Zealand is a notifiable disease, there are some variations in diagnoses that may affect the identification, classification and characterization of outbreaks and implicated cases (Shirley *et al*., 2012). For instance, the infection may be asymptomatic but meeting the clinical description in New Zealand requires the patient to show compatible symptoms accompanied by laboratory definitive evidence. Localized outbreaks can be missed by routine surveillance and/or sporadic cases (including infected individuals with no access to a local GP) being part of unrecognized outbreaks and likely unreported (Tam *et al*., 2012; Briggs *et al*., 2014). Additionally, most pathology laboratories in New Zealand used antigen and microscopy-based techniques for the diagnosis of cryptosporidiosis at the time of the study period, which are 83.7% less sensitive and 98.9% less specificity compared to PCR (Morgan *et al*., 1998; Mergen *et al*., 2020), and can overlook positive outbreak samples. Nevertheless, diagnostic laboratories are starting to switch to multiplexed qPCR (Hayman *et al*., 2020). The change among laboratories to molecular techniques will improve control programmes. However, the availability and value of subtyping provide a more in-depth and robust understanding of cryptosporidiosis epidemiology (e.g. the epidemiological interpretation of subtype occurrence and distribution trends), sources and transmission of outbreaks, and potentially, outbreaks detection to public health agencies. In the future, the application of whole-genome sequencing will likely allow population-based screening of *Cryptosporidium* species/subtypes and advance our understanding of cryptosporidiosis even further.

## Data Availability

The nucleotide sequences of the partial gp60 gene were deposited in the GenBank database under accession numbers that can be found in Garcia-R et al., 2017; Garcia-R et al., 2020.

## Supplementary material

For supplementary material accompanying this paper visit https://doi.org/10.1017/S0031182023000288. click here to view supplementary material
