# Supplementary material for: A review and analysis of cryptosporidiosis outbreaks in New Zealand
Source: Parasitology. 2023 Mar 20;150(7):606–11. doi: 10.1017/S0031182023000288 (PMC10260297; doi:10.1017/S0031182023000288)
Supplement: Supplementary file 1 [file S0031182023000288sup001.docx]

Table S1. Indel spectrums identified by TIDE analyses of the 2021 Taranaki outbreak sequences with p-value <0.001.

| Sample number | Position relative to the control |
| --- | --- |
| 19372 | -3, +2, +3 |
| 19373 | -3, +1, +2 |
| 19374 | -1, -3, -6, +3 |
| 19376 | -6, -3, -1, +3 |
| 19039 | +3, +6 |
| 19040 | -3, -6 |
| 19462 | -1, -4, +2, +3, +4 |
| 19493 | -1, -4, +2, +3, +4 |
